# Absence of Association between a Long COVID and Severe COVID-19 Risk Variant of *FOXP4* and Lung Cancer

**DOI:** 10.3389/fgene.2023.1258829

**Published:** 2023-10-26

**Authors:** Yu-Si Luo, Ke Zhang, Zhong-Shan Cheng

**Affiliations:** ^1^ Department of Emergency ICU, The Affiliated Hospital of Guizhou Medical University, Guiyang, Guizhou, China; ^2^ The Key and Characteristic Laboratory of Modern Pathobiology, The Department of Human Parasitology, School of Basic Medical Sciences, Guizhou Medical University, Guiyang, Guizhou, China; ^3^ Center for Applied Bioinformatics, St. Jude Children’s Research Hospital, Memphis, TN, United States

**Keywords:** long COVID, severe COVID-19, SNP, *FOXP4*, lung cancer, rs9367106, GWAS

## Introduction

A recent paper titled “Genome-wide Association Study of Long COVID” (Lammi et al., 2023) has been published on the preprint server medRxiv. This groundbreaking study conducted a genome-wide association study (GWAS) on long COVID, analyzing data from approximately 6.5 thousand long COVID cases and from over 1 million population controls across 24 studies spanning 16 countries. It stands as the first and largest GWAS of long COVID to date. The study identified one independent association signal represented by a single nucleotide polymorphism (SNP) known as rs9367106, which exhibited an odds ratio of 1.65. This SNP was found to be mapped to the gene *FOXP4*. This remarkable discovery suggests that individuals infected with SARS-CoV-2 who also carry the rs9367106 SNP have a 1.65-fold increased likelihood of experiencing long COVID (Lammi et al., 2023).

The identified long COVID marker, rs9367106, gains robust support due to its potential functional impact on the expression of *FOXP4*. In their study, the authors used a proxy SNP (rs12660421) closely linked to rs9367106 to represent the significant association between rs9367106 and elevated *FOXP4* expression in lung tissue according to data from the Genotype-Tissue Expression database (GTEx) (GTEx-Consortium, 2020). Furthermore, single-cell expression data from lung tissues provided additional confirmation of the potential roles of *FOXP4* in immune and alveolar cells within the lung. These pieces of evidence align coherently with our understanding, given that the lung serves as the primary target for SARS-CoV-2 infection. The presence of severe inflammation in the lung and the subsequent chronic lung damage resulting from it would be likely to contribute significantly to the development of long COVID.

However, because *FOXP4* has been reported to be linked to lung cancer (Yang et al., 2015), it remains uncertain whether the long COVID risk SNP is also connected to lung cancer. The present study endeavors to explore the possible relationship between rs9367106 and long COVID, severe COVID-19, and lung cancer. Furthermore, it aims to ascertain whether the association between rs9367106 and both long COVID and severe COVID-19 is influenced by its connection to lung cancer.

## Materials and methods

### Summary statistics of long COVID GWAS

Lammi et al. (2023) have generously provided download links for the summary statistics of four distinct long COVID GWASs. These GWASs were conducted with slightly varying case-control designs, with the criterion of retaining only SNPs having a minor allele frequency of ≥1%. The specifics of these four long COVID GWASs, along with their respective download links, are outlined below, with only the long COVID GWAS that compared strict cases of long COVID after test-verified SARS-CoV-2 infection to general population controls showing only one independent genome-wide significant hit (*P* < 5.0 × 10^−8^):

Strict cases of long COVID after test-verified SARS-CoV-2 infection (*n* = 3,018) vs. general population controls (*n* = 994,582), with its download link provided as (https://my.locuszoom.org/gwas/192226/?token=09a18cf9138243db9cdf79ff6930fdf8).

Broad long COVID cases identified as infected by any SARS-CoV-2 virus (*n* = 6,450) vs. general population controls (*n* = 1,093,995), with its download link provided as (https://my.locuszoom.org/gwas/826733/?token=c7274597af504bf3811de6d742921bc8).

Strict long COVID cases defined (*n* = 2,975) vs. strict controls restricted to individuals who were infected by SARS-CoV-2 but were not diagnosed with long COVID (*n* = 37,935): with its download link provided as (https://my.locuszoom.org/gwas/793752/?token=0dc986619af14b6e8a564c580d3220b4).

Broad long COVID cases defined (*n* = 6,407) vs. strict controls as defined in (*n* = 46,208): with its download link provided as (https://my.locuszoom.org/gwas/91854/?token=723e672edf13478e817ca44b56c0c068).

### Summary statistics of severe COVID-19 GWAS from the host genetics initiative (HGI)

Our study also incorporated five severe COVID-19 GWASs, encompassing mixed populations and ancestry-specific populations. These severe COVID-19 GWASs are accessible for download from the HGI website (COVID-19 GWAS meta-analyses round 7, release date: 8 April 2022). These GWASs investigated severe respiratory-confirmed COVID-19 in comparison to the general population across mixed and ancestry-specific subpopulations, including European (EUR), East Asian (EAS), South Asian (SAS), and African (AFR) populations. Only SNPs that met the criteria of a minor allele frequency (MAF) ≥ 1% and an imputation score > 0.6 were selected for subsequent analysis.

The severe COVID-19 GWASs involved in this study feature the following sample sizes and associated download links:

A2_ALL for mixed populations (cases = 18,152; controls = 1,145,546), with the following download link: https://storage.googleapis.com/covid19-hg-public/freeze_7/results/20220403/main/sumstats/COVID19_HGI_A2_ALL_leave_23andme_20220403_GRCh37.tsv.gz.

A2_AFR for populations with African ancestry (cases = 628; controls = 12,568), with the following download link: https://storage.googleapis.com/covid19-hg-public/freeze_7/results/20220403/pop_spec/sumstats/COVID19_HGI_A2_ALL_afr_leave23andme_20220403_GRCh37.tsv.gz.

A2_EAS for populations with East Asian ancestry (cases = 794; controls = 4,862), with the following download link: https://storage.googleapis.com/covid19-hg-public/freeze_7/results/20220403/pop_spec/sumstats/COVID19_HGI_A2_ALL_eas_leave23andme_20220403_GRCh37.tsv.gz.

A2_EUR for populations with European ancestry (cases = 13,769; controls = 1,072,442), with the following download link: https://storage.googleapis.com/covid19-hg-public/freeze_7/results/20220403/pop_spec/sumstats/COVID19_HGI_A2_ALL_eur_leave23andme_20220403_GRCh37.tsv.gz.

A2_SAS for populations with South Asian ancestry (cases = 980; controls = 47,696), with the following download link: https://storage.googleapis.com/covid19-hg-public/freeze_7/results/20220403/pop_spec/sumstats/COVID19_HGI_A2_ALL_sas_leave23andme_20220403_GRCh37.tsv.gz.

### Summary statistics of self-reported lung cancer GWAS from UK Biobank (UKB)

The self-reported lung cancer GWAS data from UK Biobank was obtained to perform a comparative analysis of association signals around the *FOXP4* locus with both the long COVID GWAS of mixed ancestries and the severe COVID-19 GWAS of European ancestry. The self-reported lung cancer GWAS was generously provided by Neale’s lab (GWAS round 2: http://www.nealelab.is/uk-biobank), and the GWAS dataset consists of 360,938 controls and 203 cases, which is publicly accessible via the following link:

https://broad-ukb-sumstats-us-east-1.s3.amazonaws.com/round2/additive-tsvs/20001_1001.gwas.imputed_v3.both_sexes.tsv.bgz.

### Analysis procedures and related scripts

Following the acquisition of these long COVID GWAS summary statistics, a custom Perl script was employed to merge the association signals of each SNP by rsID across the four long COVID GWAS summary statistics. This procedure resulted in a final table where each row is specific to a single SNP, with corresponding columns housing the summary statistics extracted from the four GWASs. To reduce file size, this merged table was subsequently compressed into a “gz” file format using 7zip. It was then uploaded to the workspace of the freely accessible online software SAS OnDemand for Academics. Within this platform, the integrated data underwent comprehensive analysis alongside summary statistics from severe COVID-19 GWASs, which were downloaded from the HGI website.

To facilitate the analysis and visualization of association signals related to both long COVID and severe COVID-19 around the *FOXP4* gene, a custom SAS script leveraging the SAS package COVID19_GWAS_Analyzer was developed. This script automates the extraction of *FOXP4* SNPs from the aforementioned GWASs and visualizes their association signals in a localized Manhattan plot.

The COVID19_GWAS_Analyzer package is openly accessible on GitHub (https://github.com/chengzhongshan/COVID19_GWAS_Analyzer), where the corresponding SAS codes for extracting *FOXP4* SNPs from these GWASs and generating the local Manhattan plot can be found at this link (https://github.com/chengzhongshan/COVID19_GWAS_Analyzer/tree/main/LongCOVID_data_and_scripts/Evaluate_FOXP4_SNPs_with_both_long_COVID_and_severe_COVID.sas).

Details for the analysis process were divided into four distinct steps. First, the merged long COVID GWASs were screened for association signals of SNPs within the genomic region chr6:41000000–42000000 (hg38), where the *FOXP4* gene resides. Second, the severe COVID-19 GWAS summary statistics for mixed populations and ancestry-stratified populations were downloaded and queried for SNPs in the same genomic region as *FOXP4*. Third, the association signals of these SNPs from both long COVID GWASs and severe COVID-19 GWASs were merged using SNP rsIDs. The resulting table was employed to visualize the association signals of these SNPs in a local Manhattan plot via the COVID19_GWAS_Analyzer. Finally, we employed a similar approach to integrate GWAS signals related to self-reported lung cancer around the *FOXP4* loci with those of long COVID and severe COVID-19 within the European population. We conducted a specific examination of the association between the target SNP, rs9367106, and self-reported lung cancer. Additionally, we identified 20 SNPs displaying suggestive association signals (*P* < 5 × 10^−4^) in the vicinity of the *FOXP4* locus in relation to self-reported lung cancer. These selected SNPs underwent expression quantitative trait locus (eQTL) analysis using the GTEx API (https://gtexportal.org/api/v2/redoc#tag/GTEx-Portal-API-Info), and COVID19_GWAS_Analyzer implemented a SAS Macro to facilitate the automation of the eQTL analysis.

All the analyses described above were executed within the SAS On Demand for Academics. Additionally, the customized Perl script used for merging the association signals of each SNP by rsID across the four long COVID GWAS summary statistics is available on GitHub (https://github.com/chengzhongshan/COVID19_GWAS_Analyzer/tree/main/LongCOVID_data_and_scripts/MergeBigFiles.PL).

## Results

### rs9367106 of *FOXP4* associated with both long COVID and severe COVID-19

**FIGURE 1:**
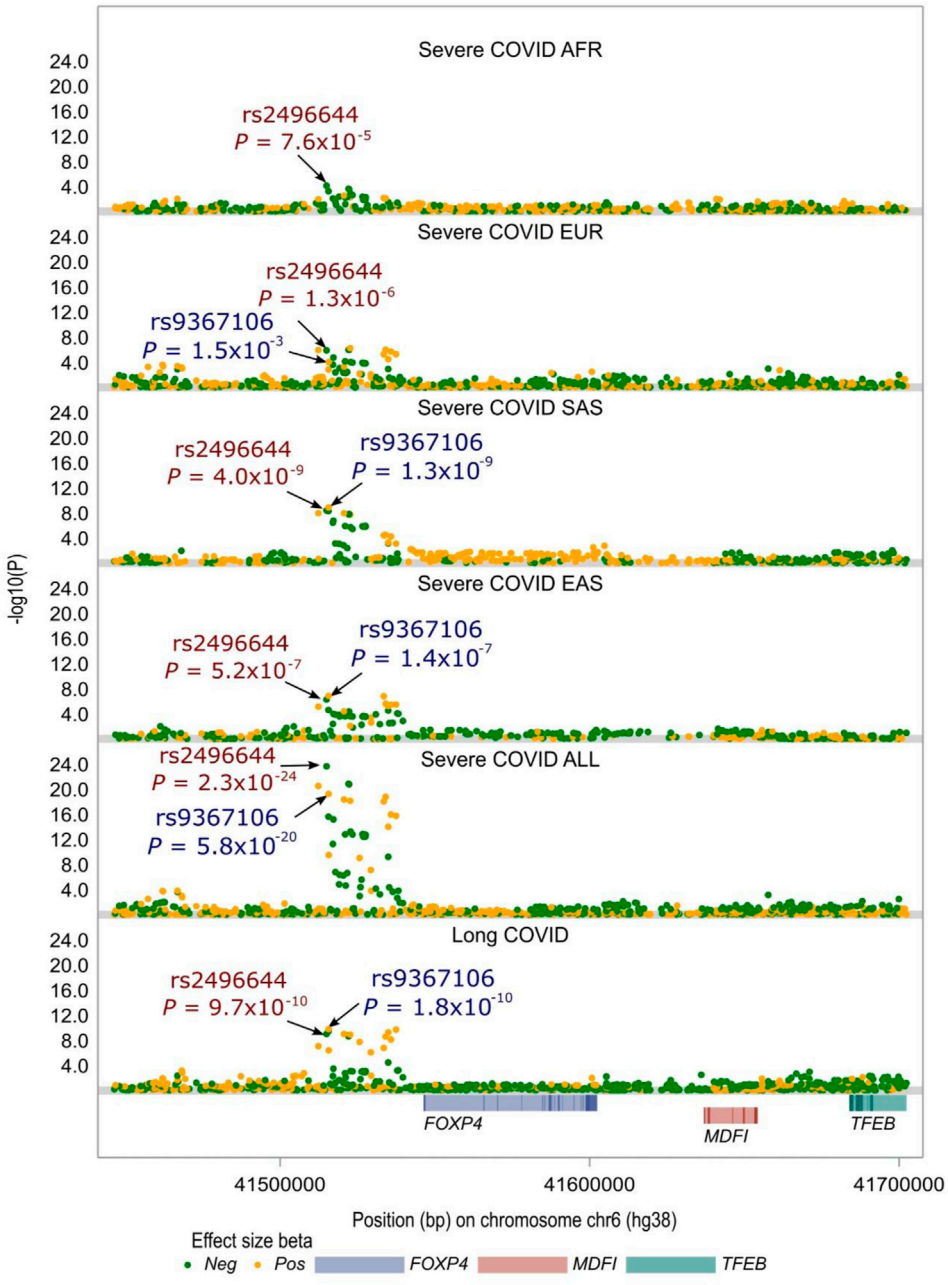
Local Manhattan plot of association signals around the *FOXP4* locus across severe COVID-19 GWASs and long COVID GWAS. rs9367106 is the most prominent independent SNP demonstrating genome-wide significance in the long COVID GWAS. This SNP also shows genome-wide significance, although with a somewhat weaker association signal compared to the top SNP, rs2496644, identified in severe COVID-19 among mixed populations (Severe COVID ALL). Note: the severe COVID-19 GWAS of African populations (Severe COVID AFR) does not include rs9367106, and arrows have been employed to highlight the presence of these two SNPs in the long COVID GWAS and five other severe COVID-19 GWAS datasets, with the genome-wide significance threshold set at *P* < 5 × 10^−8^. Further details regarding these GWASs and associated analyses can be found in the Materials and Methods section.

The *FOXP4* variant, rs9367106, was strongly associated with both long COVID and severe COVID-19 outcomes. Notably, the authors reported a significant association of this SNP with long COVID, as depicted in Figure 1. To have a deep understanding of this finding, we merged long COVID GWAS data with the most recent severe COVID-19 GWAS data shared by the HGI (refer to Materials and Methods). Our analysis affirmed that, across a diverse range of populations, including a mixed population and four major populations stratified by ancestry (European—EUR, African—AFR, East Asian—EAS, and South Asian—SAS), rs9367106 was consistently identified as a genome-wide significant hit (*P* < 5.0 × 10^−8^) in the GWAS of severe COVID-19. This association also held true for the mixed population as well as the South Asian—SAS population, with a somewhat weaker association observed in the East Asian—EAS population, nearing genome-wide significance, as illustrated in Figure 1. Furthermore, rs9367106 exhibited a strong linkage disequilibrium with the top SNP associated with severe COVID-19, rs2496644 (with an R^2^ value of 0.88 in the European population). Interestingly, rs2496644 demonstrated similar association signals across four GWAS datasets, encompassing the long COVID GWAS and the severe COVID-19 GWAS conducted in mixed populations, as well as in EAS and SAS populations. These findings align with the understanding that severe COVID-19 patients are more likely to develop long COVID.

### The long COVID and severe COVID-19 risk SNP rs9367106 close to the *FOXP4* gene does not associate with lung cancer

**FIGURE 2:**
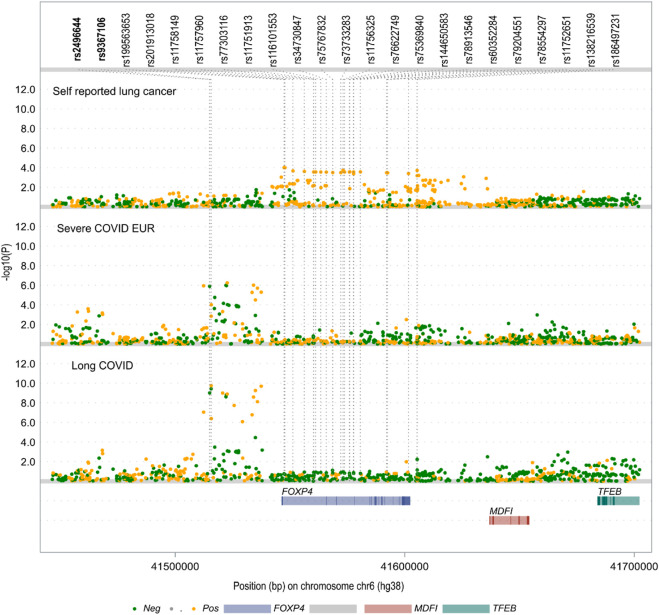
The local Manhattan plot highlights contrasting association signals at the *FOXP4* locus within the UK Biobank self-reported lung cancer GWAS when compared to both the long COVID GWAS and severe COVID-19 GWAS. The two single nucleotide polymorphisms (SNPs) highlighted in bold exhibit genome-wide significance (*P* < 5.0 × 10^−8^) in both COVID-related GWAS datasets but fail to achieve nominal significance in the context of self-reported lung cancer (both with *P*s > 0.05). Conversely, there are 20 SNPs displaying suggestive associations (*P* < 5 × 10^−4^) with self-reported lung cancer, yet they do not demonstrate significant associations (all with *P*s > 0.05) in the two COVID-related GWAS datasets. Additional details regarding the long COVID GWAS with mixed ancestries and the severe COVID-19 GWAS of the European population can be found in the Materials and Methods section.

To explore the potential relationship between rs9367106 and lung cancer, we conducted an analysis using the UK Biobank self-reported lung cancer GWAS dataset, which primarily comprises individuals of European ancestry. Additionally, we compared the association signals around the *FOXP4* gene in the self-reported lung cancer GWAS with those from the severe COVID-19 GWAS of European samples and the long COVID GWAS, which includes individuals of mixed ancestries. As illustrated in Figure 2, the association peaks near rs9367106 observed in both the long COVID GWAS and the severe COVID-19 GWAS were not present in the self-reported lung cancer GWAS. This discrepancy can be attributed mainly to two specific SNPs, along with others in close proximity, which showed no significant association with lung cancer (with *P*s > 0.05). However, it is noteworthy that 20 SNPs located within the gene body of *FOXP4* displayed suggestive associations with lung cancer (*P*s < 5 × 10^−4^). Interestingly, these SNPs did not achieve nominal significance in the two COVID-related GWAS datasets. This observation strongly suggests that different SNPs may underlie the potential associations of *FOXP4* with lung cancer, as well as with long COVID and severe COVID-19. In summary, our findings indicate that *FOXP4* is indeed implicated in lung cancer, but the long COVID and severe COVID-19 risk SNP rs9367106 does not exhibit an association with lung cancer.

### Expression quantitative trait locus (eQTL) analysis for two COVID risk SNPs and 20 SNPs of *FOXP4* that are potentially predisposing to lung cancer

**FIGURE 3:**
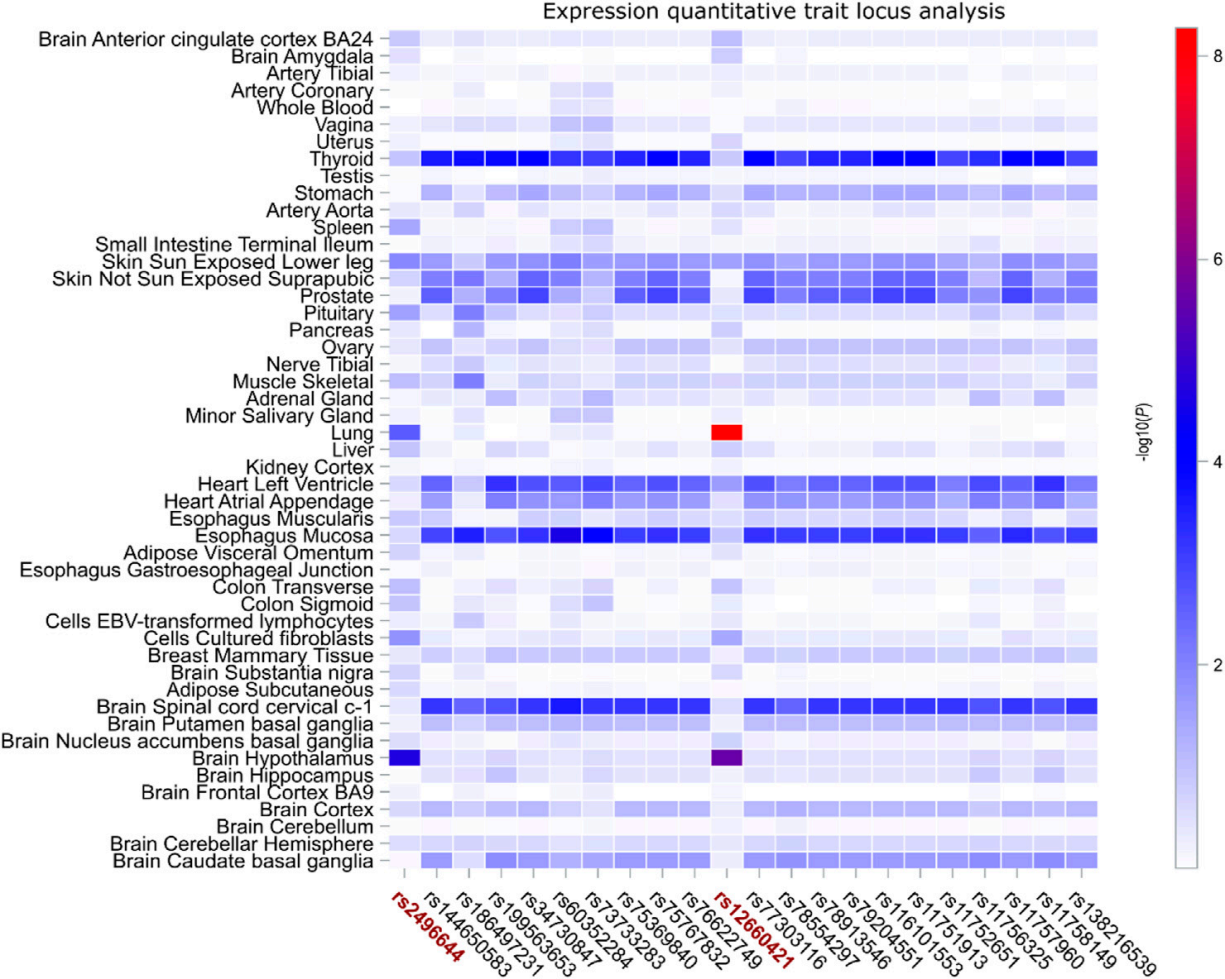
Expression quantitative trait locus (eQTL) analysis correlating *FOXP4* expression with two COVID risk SNPs and 20 SNPs potentially associated with self-reported lung cancer. The two COVID risk SNPs, rs22496644 and rs12660421, are depicted in dark red, with the latter being closely linked to rs9367106, the genotype of which was not included in the GTEx database. Remarkably, rs12660421 demonstrates the most robust association with *FOXP4* expression in both the lung and brain hippocampus tissues, while rs22496644 exhibits similar but slightly weaker eQTL signals in these two tissues. Conversely, the remaining 20 SNPs only showing suggestive associations in self-reported lung cancer do not exhibit pronounced associations with *FOXP4* expression in the lung and brain hippocampus but rather tend to function as eQTLs across various other tissues. Note: the color bar on the right side of the graph represents the unadjusted eQTL association −log10(*P*) values.

To further substantiate our hypothesis regarding the regulatory influence of these two COVID risk SNPs, rs9367106 and rs2496644, along with the 20 SNPs potentially associated with lung cancer, on *FOXP4* expression, we conducted an extensive eQTL analysis across 49 GTEx tissues. This analysis involved correlating the genotypes of each SNP with *FOXP4* expression in each GTEx tissue. Given the absence of rs9367106 in GTEx, we focused on its highly linked SNP, rs12660421. Our findings revealed that both COVID risk SNPs, rs2496644 and rs12660421, exhibited robust eQTL effects on *FOXP4* expression in the lung and brain hippocampus tissues. Notably, the lung tissue displayed the most pronounced association between *FOXP4* expression and rs12660421 (as illustrated in Figure 3). In contrast, these 20 SNPs potentially associated with lung cancer did not exhibit eQTL effects in the lung and brain hippocampus tissues. However, these SNPs displayed consistent eQTL associations across various other tissues, including the thyroid, left ventricle of the heart, esophagus mucosa, and cervical region of the spinal cord. In conclusion, the observation of these SNPs displaying distinct eQTL patterns in different tissues suggests that diverse genetic elements within *FOXP4* specifically contribute to COVID or lung cancer by influencing *FOXP4* expression across different tissues.

## Discussion

The long COVID GWAS has illuminated a compelling connection between a genetic marker proximal to the *FOXP4* gene and the incidence of long COVID. The potential involvement of *FOXP4* in the development of long COVID finds support through multiple layers of evidence, including expression quantitative trait locus analysis, single-cell gene expression profiling, and epigenetic investigations. However, two noteworthy limitations merit discussion.

First, distinguishing whether the identified SNP or *FOXP4* itself is more linked to severe COVID-19 or long COVID posed a challenge due to the distinct clinical profiles of two COVID phenotypes. Lammi et al. (2023) attempted to address this by employing Mendelian randomization (Davis et al., 2023) to explore the causal relationship between COVID-19 hospitalization and long COVID by excluding overlapping samples used in both GWASs. However, since these samples originated from the same source (i.e., HGI), potential confounding factors might have a similar prevalence in these shared samples. This limitation is underscored by reports of *FOXP4* variants being associated with lung cancer (Yang et al., 2015). In our analysis, we identified different *FOXP4* regulatory variants that were associated with long COVID/severe COVID-19 or lung cancer but not all of these traits at the same time. One possible explanation for these findings is that other underlying factors, instead of lung cancer, could influence the association between *FOXP4* variants and both severe COVID-19 and long COVID. Further association tests considering additional covariates may clarify these associations.

Second, SNP associations with long COVID varied among populations, emphasizing the need for confirmation through further investigation. The SNP (rs9367106) displayed varying minor allele frequencies across populations, with the lowest in Europeans and higher frequencies in Asian populations. It showed nominal significance in only a few sub-cohorts used for the meta-analyses of long COVID GWAS (Lammi et al., 2023), indicating the required validation in diverse populations.

It is essential to emphasize that the relationship between long COVID and other COVID phenotypes is intricately intertwined. Long COVID represents a broad phenotype encompassing persistent post-COVID symptoms, which endure for more than 6 months following SARS-CoV-2 infection (Davis et al., 2023). Researchers are still actively working to define the key symptoms that can accurately characterize long COVID. The current long COVID GWAS did not encompass all the key long COVID symptoms (Davis et al., 2023) from affected patients. Future GWAS efforts may focus on more cohesive long COVID phenotypes or sub-phenotypes.

Despite limitations, Lammi et al. (2023)’s findings represent a significant breakthrough. With the complexity of long COVID symptoms, future genetic studies are expected to uncover more associations related to this condition.
